# A case of testicular tumor and respiratory failure caused by choriocarcinoma syndrome managed through modified chemotherapy and extracorporeal membrane oxygenation

**DOI:** 10.1002/iju5.12725

**Published:** 2024-04-08

**Authors:** Tetsuro Shiraiwa, Hiromichi Katayama, Yudai Iwasaki, Shingo Kimura, Yohei Satake, Takuma Sato, Yoshihide Kawasaki, Naoki Kawamorita, Shinichi Yamashita, Akihiro Ito

**Affiliations:** ^1^ Department of Urology Tohoku University Graduate School of Medicine Sendai Miyagi Japan; ^2^ Department of Anesthesiology and Perioperative Medicine Tohoku University Graduate School of Medicine Sendai Miyagi Japan

**Keywords:** choriocarcinoma syndrome, extracorporeal membrane oxygenation, reduced chemotherapy, respiratory failure, resticular tumor

## Abstract

**Introduction:**

Choriocarcinoma syndrome with multiple lung metastases has a poor prognosis and causes respiratory failure due to alveolar hemorrhage. We encountered a case where the introduction of extracorporeal membrane oxygenation effectively sustained oxygenation until chemotherapy took effect on lung metastases of testicular tumors.

**Case presentation:**

A 35‐year‐old man with dyspnea was referred to our hospital. He showed left testicular tumor with multiple lung metastases. Serum human chorionic gonadotropin level was also elevated. Reduced chemotherapy was initiated and extracorporeal membrane oxygenation was administered because of low oxygen levels on the fourth day. Chemotherapy successfully reduced the size of the lung masses, and extracorporeal membrane oxygenation was discontinued. Respiratory status improved substantially, but the patient died of brain metastases 4 months later.

**Conclusion:**

Extracorporeal membrane oxygenation may be a useful option for managing respiratory failure resulting from choriocarcinoma syndrome until the respiratory condition is improved by chemotherapy for testicular tumors.

**Keynote message**

Respiratory failure due to choriocarcinoma syndrome of testicular tumor is an oncologic emergency condition. It is important to maintain oxygenation until chemotherapy is successful, and ECMO may be useful if oxygenation cannot be maintained with ventilatory management.

**Abbreviations & Acronyms**
ARDS: acute respiratory distress syndrome
CT: computed tomography
EAU: European Association of Urology
ECMO: extracorporeal membrane oxygenation
EP: etoposide, cisplatin
hCG: human chorionic gonadotropin
ICU: intensive care unit
IGCCC: International Germ Cell Consensus Classification
TIP: paclitaxel, ifosfamide, cisplatin
VIP: etoposide, ifosfamide, cisplatin

## Introduction

Choriocarcinoma syndrome is characterized by highly elevated hCG levels and bleeding from metastase sites.^1^ The lungs are the typical sites of hemorrhage, and extensive multiple lung metastases combined with ventilatory disturbances due to alveolar hemorrhage can lead to severe respiratory failure with an extremely poor prognosis.^2^ Herein, we report a case of a testicular tumor with respiratory failure due to choriocarcinoma syndrome where ECMO was used to sustain oxygenation and facilitate the continuation of chemotherapy.

## Case report/case presentation

**Fig. 1:**
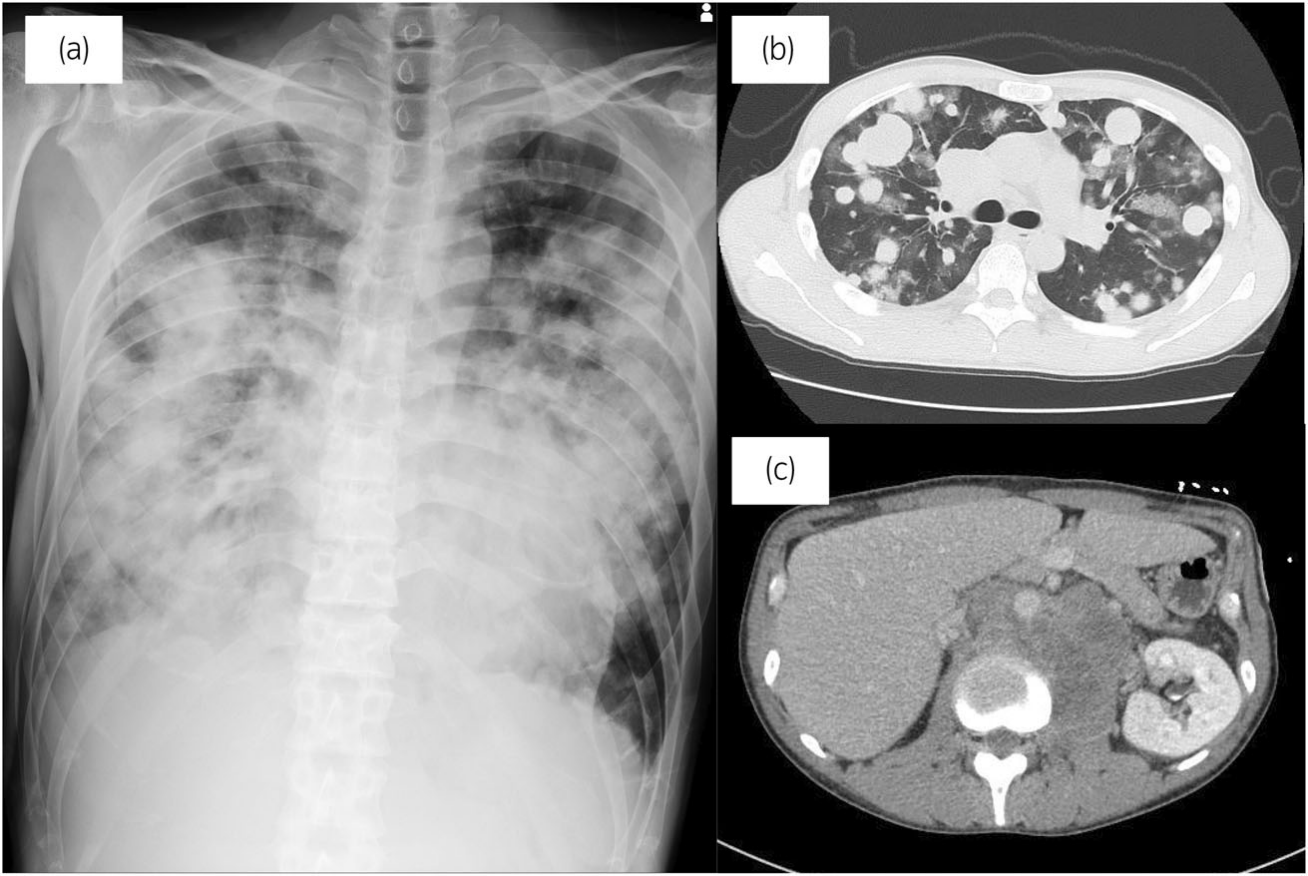
Image before treatment. (a) Chest X‐ray showed extensive infiltrative shadows in the lung fields. (b) Chest CT scan showed multiple nodular shadows with ground‐glass opacities suggestive of hemorrhage in the lung. (c) Abdominal contrast‐enhanced CT scan showed enlarged retroperitoneal lymph nodes.

A 35‐year‐old man noticed back pain 2 weeks before presentation and developed dyspnea. He had no relevant medical history or family history. When he visited the hospital, he presented with tachypnea. Blood gas analysis showed hypoxemia with a PaO2 of 81 mmHg under 10 L/min oxygen mask administration. CT showed multiple lung tumors and enlarged para‐aortic lymph nodes. Ultrasonography revealed a left testicular tumor. He was referred to our hospital on the same day and immediately admitted to the ICU. Testicular tumor marker levels were elevated: hCG, 197,619 mIU/mL; alpha‐fetoprotein, 7562 ng/mL; and lactate dehydrogenase, 2808 U/L. Chest radiography revealed extensive infiltrative shadows in the lungs (Fig. 1a). Contrast‐enhanced CT scan revealed extensive multiple nodular shadows with ground‐glass opacities suggestive of lung hemorrhage (Fig. 1b). The retroperitoneal lymph nodes in the abdomen were enlarged (Fig. 1c). Brain metastases were not observed. The patient was diagnosed with clinical stage IIIC (TXN3M1aS3) nonseminoma testicular germ cell tumor according to the tumor, node, and metastasis classification.^3^ He also experienced respiratory failure due to choriocarcinoma syndrome, pulmonary metastasis, and alveolar hemorrhage. He was single, had a partner, and hoped to raise a child in the future. However, due to his poor general condition, Sperm cryopreservation was not performed. He was deemed to require urgent systemic treatment, and the histological diagnosis with high orchidectomy was omitted.

**Fig. 2:**
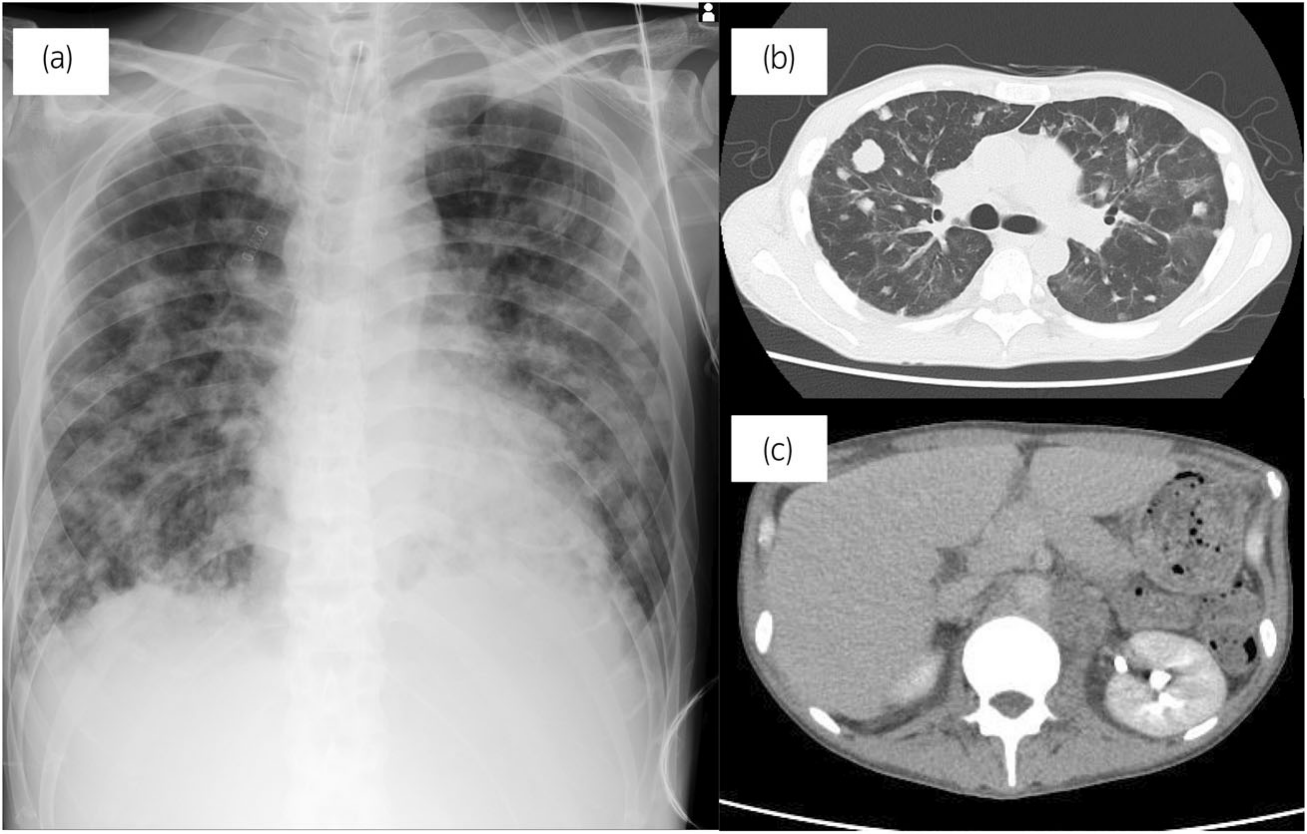
Image at the discharge from ICU. Chest X‐ray (a) and CT (b) Infiltration shadows in the lung fields decreased, compared with those before chemotherapy. (c) Abdominal contrast‐enhanced CT scan showed reduction of retroperitoneal lymph nodes.

**Fig. 3:**
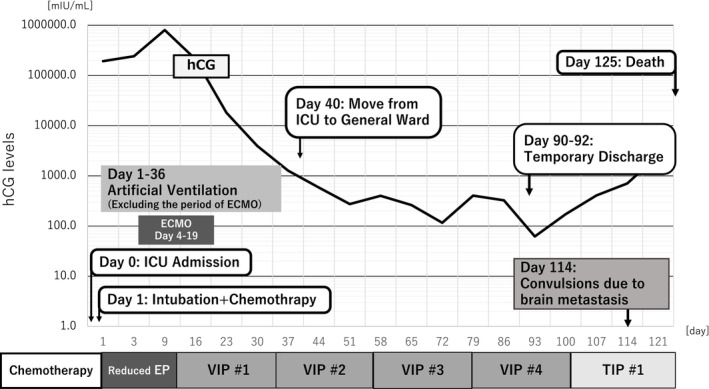
Time course and change in serum hCG levels.

Reduced EP therapy (etoposide, 100 mg/m^2^ and cisplatin, 20 mg/m^2^ on days 1–3) was started the day after admission to the ICU (day 1), and endotracheal intubation and ventilatory management were performed on the same day. Standard induction chemotherapy followed by reduced EP therapy according to previous reports.^4^ Bleeding in the airway increased, and it was difficult to maintain oxygenation using an artificial ventilator with positive pressure ventilation. ECMO was initiated on day 4. Positive pressure ventilation was discontinued during ECMO. Induction chemotherapy using VIP (etoposide, 75 mg/m^2^; ifosfamide, 1200 mg/m^2^; and cisplatin, 20 mg/m^2^ on days 1–5) was initiated under ECMO on day 11. In addition, the intensivists removed blood sputum multiple times a day using bronchoscopy until day 15. ECMO was discontinued on day 19 because airway bleeding decreased and chest radiography images and oxygenation improved substantially. On day 30, the hCG level decreased to 1271 mIU/mL, and CT showed shrinkage of the lung masses and retroperitoneal lymph nodes (Fig. 2a–c). His respiratory condition stabilized and the tracheal tube was removed on day 36. On day 40, the patient was moved from the ICU to the general ward. After four courses of full‐dose VIP therapy, the patient was temporarily discharged on day 90 and spent several days at his home. However, the hCG level did not decrease to the normal level, and he underwent salvage chemotherapy using TIP (paclitaxel, 175 mg/m^2^ on day 1; ifosfamide, 1000 mg/m^2^ on days 1–5; and cisplatin, 20 mg/m^2^ on days 1–5) (Fig. 3). Before completing two cycles of TIP, he experienced convulsions and loss of consciousness. He died of brain hemorrhage and edema attributed to multiple brain metastases, 4 months following treatment initiation.

## Discussion

Choriocarcinoma syndrome is a life‐threatening condition characterized by bleeding that occurs alongside rapid tumor growth containing choriocarcinoma components or the rapid breakdown of tumor cells due to chemotherapy. Regarding the timing of choriocarcinoma syndrome, it typically develops after chemotherapy, but its onset before treatment has also been reported.^5^ The treatment of choriocarcinoma syndrome with respiratory failure is challenging, and the key is to maintain oxygenation and continue chemotherapy.^2^ In the present case, the respiratory failure occurred before chemotherapy and worsened after induction chemotherapy. The patient's respiratory failure improved with the introduction of ECMO in addition to reducing the initial chemotherapy dose.

According to the IGCCC, four cycles of bleomycin, etoposide, cisplatin or VIP regimens are recommended as standard induction chemotherapy for patients with poor prognosis.^6^ However, when lung metastases are extensive, as in this case, full‐dose standard chemotherapy carries the risk of alveolar hemorrhage due to tumor collapse. Massard *et al*. reported that reduction of dose at the first cycle of chemotherapy reduced the risk of death due to acute respiratory distress syndrome (ARDS) in patients with testicular cancer that had metastasized to the lungs and caused respiratory failure.^4^ To address these issues, the 2023 European Association of Urology (EAU) guidelines indicate that patients with extensive lung metastases should be treated with reduced EP therapy (CDDP reduced dose from 5 to 3 days) to reduce the risk of ARDS. The EAU guidelines also state that standard chemotherapy should be started after the ARDS risk has passed, typically after 10 days. This guideline was written to prevent respiratory failure due to choriocarcinoma syndrome after initiation of therapy. However, it was difficult to determine the content and timing of the chemotherapy regimens for patients with pretreatment respiratory failure. In this case, ECMO was introduced due to worsening of ARDS on day 4 of treatment. On day 11 of treatment, respiratory failure due to alveolar hemorrhage had not improved, but oxygenation was maintained by ECMO. His condition was not expected to improve by delaying standard chemotherapy, and treatment was initiated the same day. Because of the severe respiratory failure, a regimen containing bleomycin, which is a pulmonary toxicant, was not selected.

In this case, ECMO was introduced because ventilatory management via intubation could not maintain sufficient oxygenation. ECMO has the advantage of ensuring breathing by extracorporeal circulation using artificial lungs, allowing oxygenation without positive pressure ventilation, and preventing tissue damage caused by high concentrations of oxygen, thus allowing lung rest.^7^ However, problems include the risk of hemoptysis and bleeding due to infection, the physical destruction of blood cells in the circuit, and the need for heparinization to prevent circuit obstruction. The advantages and disadvantages of ECMO in cancer‐bearing patients requiring chemotherapy have been debated because of the difficulties in controlling bleeding and infection. Recent case reports and retrospective studies, although limited, have reported the effectiveness of ECMO under conditions of good sensitivity to chemotherapy after examining cancer type and patient characteristics.^8^ Although there have been reports of temporary improvement in the respiratory status after ECMO in testicular tumors with multiple lung metastases,^9^ this is the first case in which ECMO was effective for the management of respiratory failure due to choriocarcinoma syndrome in testicular tumors. The patient died of bleeding from brain metastases; however, he was temporarily discharged from the hospital and spent several days at his home comfortably until the next treatment began. Therefore, because testicular tumors are highly sensitive to chemotherapy, proactive consideration of ECMO is warranted, particularly when positive pressure ventilation proves inadequate in sustaining oxygenation, with special attention to cases involving brain metastases.

## Conclusion

ECMO may be useful for treating respiratory failure due to choriocarcinoma syndrome in testicular tumors. When oxygenation cannot be maintained with positive pressure ventilation in choriocarcinoma syndrome of testicular tumors, ECMO should be considered to maintain oxygenation and continue chemotherapy.

## Author contributions

Tetsuro Shiraiwa: Writing – original draft. Hiromichi Katayama: Conceptualization; writing – review and editing. Yudai Iwasaki: Investigation. Shingo Kimura: Data curation. Yohei Satake: Data curation. Takuma Sato: Data curation. Yoshihide Kawasaki: Data curation. Naoki Kawamorita: Data curation. Shinichi Yamashita: Writing – review and editing. Akihiro Ito: Supervision.

## Conflict of interest

The authors declare no conflict of interest.

## Approval of the research protocol by an Institutional Reviewer Board

The study protocol was approved by the Ethics Committee of Tohoku University Hospital (Approval no. 34338).

## Informed consent

Written informed consent was obtained from the patient's parents for publication of this case report and any accompanying images.

## Registry and the Registration No. of the study/trial

Not applicable.

## Data Availability

All data generated or analyzed during this study are included in this article. Further inquiries can be directed to the corresponding authors.
